# Recent Progress on Advanced Flexible Lithium Battery Materials and Fabrication Process

**DOI:** 10.3390/nano14221856

**Published:** 2024-11-20

**Authors:** Mi Zhou, Daohong Han, Xiangming Cui, Jingzhao Wang, Xin Chen, Jianan Wang, Shiyi Sun, Wei Yan

**Affiliations:** 1 Department of Environmental Science and Engineering, State Key Laboratory of Multiphase Flow in Power Engineering, School of Energy and Power Engineering, Xi’an Jiaotong University, Xi’an 710049, China; zhoumi2030@stu.xjtu.edu.cn (M.Z.); handaohong@stu.xjtu.edu.cn (D.H.);; 2 School of Chemistry, Xi’an Jiaotong University, Xi’an 710049, China

**Keywords:** wearables, flexible electrodes, flexible material processing technology, flexible structure design, carbon materials

## Abstract

Flexible energy storage devices have attracted wide attention as a key technology restricting the vigorous development of wearable electronic products. However, the practical application of flexible batteries faces great challenges, including the lack of good mechanical toughness of battery component materials and excellent adhesion between components, resulting in battery performance degradation or failure when subjected to different types of deformation. It is imperative to develop flexible batteries that can withstand deformation under different conditions and maintain stable battery performance. This paper reviews the latest research progress of flexible lithium batteries, from the research and development of new flexible battery materials, advanced preparation processes, and typical flexible structure design. First, the types of key component materials and corresponding modification technologies for flexible batteries are emphasized, mainly including carbon-based materials with flexibility, lithium anode materials, and solid-state electrolyte materials. In addition, the application of typical flexible structural designs (buckling, spiral, and origami) in flexible batteries is clarified, such as 3D printing and electrospinning, as well as advanced fabrication techniques commonly used in flexible materials and battery components. Finally, the limitations and coping strategies in the practical application of flexible lithium batteries are discussed, which provides new ideas for future research.

## 1. Introduction

**Figure 1:**
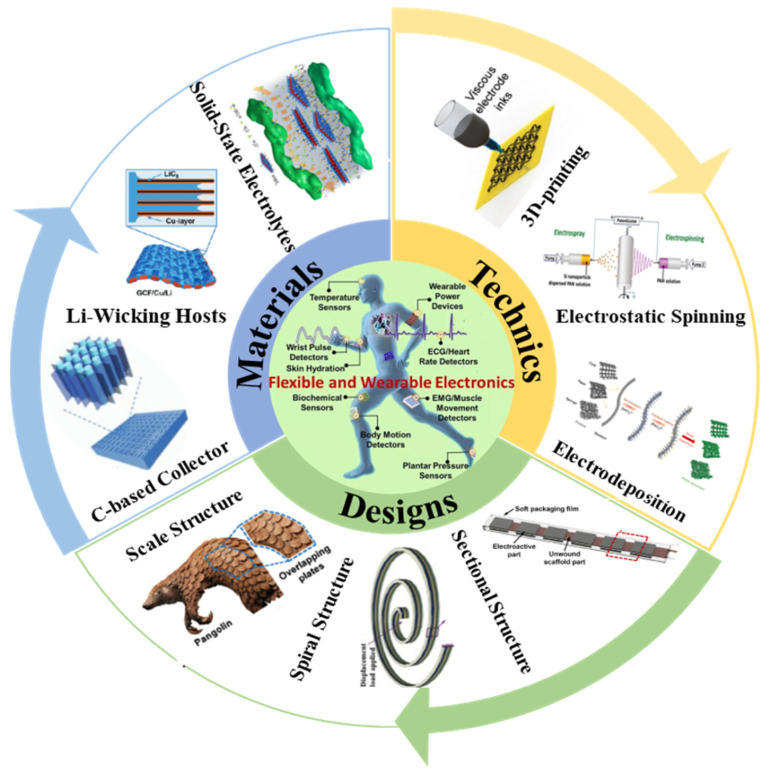
Materials, process, and structure design of flexible batteries. Materials: C-base Collector. Reprinted with permission from Reference [4]. Li-Wicking Hosts. Reprinted with permission from Reference [5]. Solid-State Electrolytes (SSE). Reprinted with permission from Reference [6]. Technics: 3DP-printing. Reprinted with permission from Reference [7]. Electrostatic Spinning. Reprinted with permission from Reference [8]. Electrodeposition. Reprinted with permission from Reference [9]. Designs: Sectional Structure. Reprinted with permission from Reference [10]. Spiral Structure. Reprinted with permission from Reference [11]. Scale Structure. Reprinted with permission from Reference [12].

With the increasing demand for wearable electronic products and portable devices, the development and design of flexible batteries have attracted extensive attention in recent years [1]. Traditional lithium-ion batteries (LIBs) usually lack sufficient mechanical flexibility to stretch, bend, and fold, thus making it difficult to achieve practical applications in the corresponding flexible electronic products [2,3]. Therefore, it is imperative to develop flexible, lightweight, and deformable energy storage devices. Currently, the main issues with flexible LIBs are: (i) electrode and electrolyte materials lack flexibility, leading to poor interlayer contact. During the battery bending process, active materials are easy to detach from the current collector, resulting in electrode cracking and battery performance degradation; (ii) The design of flexible battery packs is not sufficiently adaptable, and the individual cells within the pack suffer from low energy density. This inadequacy fails to meet the requirements for flexible deformation and high battery performance in practical applications; (iii) The large-scale production cost of flexible LIBs is high, and there is an urgent need to develop simple, rapid, and low-cost battery packaging processes for flexible electrodes and electrolytes. Therefore, the flexible structural design of battery packs is highly necessary (Figure 1). In order to overcome the above issues, the main strategies for developing flexible batteries at present include researching flexible battery materials, applying advanced material manufacturing processes, and designing flexible battery structures.

The basic strategy for the research and development of materials for flexible LIBs has been focused on the use of novel materials to enhance the mechanical flexibility of the battery components. The usual strategy is to replace rigid battery components with flexible electrode materials. For example, carbon-based materials such as carbon nanotubes (CNTs), carbon nanofibers (CNFs), carbon cloth (CC), or graphene derivatives are utilized as the electrode current collectors due to their high flexibility and high electronic conductivity [2]. The lithium metal anode can be modified to achieve superior mechanical flexibility by employing simple processing techniques or incorporating flexible 3D porous materials. In traditional LIBs, liquid electrolytes with high conductivity and good compatibility with the electrodes are typically used, along with polymer separators to prevent short circuits between the positive and negative electrodes [13]. However, considering that flexible electronic products require frequent bending during use, the use of liquid electrolytes is likely to result in electrolyte leakage and separator displacement, leading to potential safety hazards [14,15]. Therefore, in the selection and research of electrolyte materials for flexible batteries, solid-state electrolytes (SSE) are more suitable for flexible lithium batteries, offering greater safety and reliability compared to liquid electrolytes [16]. In addition, the utilization of advanced processing technology can also effectively improve the mechanical flexibility and interlayer contact of flexible battery materials. For example, 3D printing technology (3DP), electrospinning technology, coating and spraying, Electrophoretic deposition (EPD), etc., can effectively achieve a strong adhesion of active materials and current collectors to realize the integration of battery layers [17]. Ultimately, the structure of flexible batteries is crucial to accelerate the commercial applications of flexible electronic products. The current flexible battery structure is mainly classified according to the spatial structure, including one-dimensional battery structure (linear, cable and fiber type), two-dimensional battery structure (island and grid type), and three-dimensional battery structure (snake, origami, and spine type) [18,19,20]. At present, many researchers have carried out structural bionics based on the flexible materials existing in reality and have built flexible structure batteries with the characteristics of stretchable bending and folding deformation [21].

With these considerations, this work first reviews the research progress of flexible battery component materials, including the development and application of carbon-based materials and the strategies for and studies of flexible lithium metal anodes and electrolytes. Advanced flexible material processing technologies and typical flexible structure batteries are summarized and analyzed. Finally, strategies are provided for industry to realize the practical applications of flexible lithium-based batteries, and new insights are provided for future research.

## 2. Flexible Materials for Flexible LIBs

Compared with traditional LIBs, flexible batteries require flexible components, including flexible electrodes, separators, and electrolytes. Table 1 shows the structure and composition of conventional and flexible lithium batteries. Compared with the components of ordinary batteries, flexible electrodes are usually made of a flexible conductive film substrate with functional positive and negative electrode slurries. The electrolyte, on the other hand, uses a flexible solid-state electrolyte to improve the mechanical toughness and flexibility of the battery. The selection of electrode and electrolyte materials directly determines whether the flexible battery can achieve excellent electrochemical performance under different strains, which is the core issue for the realization of functionalized flexible battery. Here, the research status and flexible modification strategies of flexible carbon-based materials, lithium-based anodes, and solid-state electrolyte materials are introduced in detail.

### 2.1. Flexible Carbon-Based Materials

Flexible carbon-based materials with high specific surface area, high conductivity, and ideal chemical stability are widely used for the preparation of flexible electrodes [16,22]. The main carbon-based electrode materials used for flexible LIBs are CNTs, graphene, CNFs, and CC [16]. The flexible carbon-based materials themselves can be embedded with a certain amount of Li^+^ to be used directly as anodes or combined with other active substances to form freestanding electrodes. Moreover, they can be used in flexible current collectors, so flexible carbon-based materials can effectively avoid the use of conductive additives, adhesives, and some rigid current collectors, and realize the significant improvement of electrode flexibility [23].

#### 2.1.1. Carbon Nanotube Electrodes for Flexible LIBs

CNTs are promising one-dimensional carbon materials for flexible electrodes due to their large specific surface area, low density, chemical stability, high electrical conductivity, and mechanical flexibility [4,24]. Therefore, a large number of studies have focused on their use as active lithium storage materials and supporting substrates.

**Figure 2:**
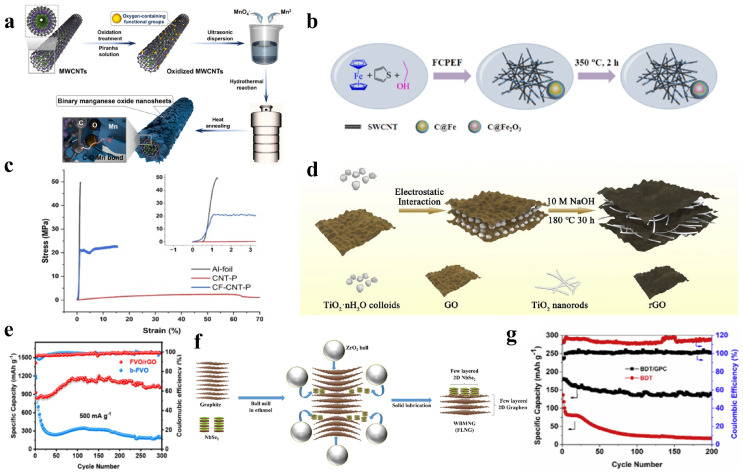
CNTs and graphene based flexible electrode materials. (**a**) The synthesis procedure of the MnO_x_/MWCNTs nanocomposites. Reprinted with permission from Reference [24]. (**b**) The preparation procedure of flexible C@Fe_2_O_3_/SWCNT membrane. Reprinted with permission from Reference [29]. (**c**) Stress-strain curves of Al-foil, CNT-P, and CF-CNT-P. Reprinted with permission from Reference [31]. (**d**) Schematic of the synthesis process of TNGC material. Reprinted with permission from Reference [39]. (**e**) Cycling performance of FVO/rGO and b-FVO materials (500 mAh g^−1^). Reprinted with permission from Reference [40]. (**f**) Schematic illustration of the synthesis process of few-layer NbSe_2_@graphene by WBM. Reprinted with permission from Reference [41]. (**g**) The cycling performance and corresponding coulombic efficiency of BDT/3DGraphene and BDT at 0.5 C. Reprinted with permission from Reference [44].

Flexible electrode materials usually need to be combined with active materials and flexible substrates to obtain high mechanical and electrochemical properties. High specific capacity Si, Ge, Sn [25], and transition metal oxides (TMOs) [26,27,28] have attracted attention as promising anode active materials for LIBs. Although these high-performance candidates can replace conventional graphite anodes as high-capacity electrode actives [23], they still suffer from some inherent defects (e.g., poor electrical conductivity) that greatly limit their future practical applications. The electrochemical performance of TMOs can be greatly improved by shrinking them to the nanoscale level and constructing layered hybrid electrodes with carbon matrix. Guo et al. successfully synthesized a series of MnO_x_/multi-walled carbon nanotubes (MWCNTs) nanocomposites with different compositions and morphologies by adjusting the additive amount of oxygen-containing functional groups on the surfaces of MWCNTs and allowing the binary manganese oxide nanosheets to grow perpendicularly and homogeneously over the oxidized MWCNTs (Figure 2a) [24]. Similarly, Wang et al. employed a novel Floating catalyst premixed ethanol flame (FCPEF) approach by injecting ethanol/ferrocene/thiophene (EFT) solution into an evaporator for gasification, which was then carried by Ar and O_2_ into a low-pressure pre-mixed ethanol flame (C_2_H_5_OH-O_2_-Ar), and finally in situ synthesized single-walled carbon nanotube (SWCNT) network decorated with a carbon-encapsulated Fe_2_O_3_ nanoparticles (C@Fe_2_O_3_/SWCNT) membrane (Figure 2b) [29]. The interconnected conductive network of SWCNT improved the transport of Li^+^ and electrolyte penetration and buffered the volume changes of anode during lithiation/delithiation. As a result, C@Fe_2_O_3_/SWCNT exhibited a high capacity (1294.7 mAh g^−1^ at 50 mA g^−1^) and a high cycling stability (563.7 mAh g^−1^ after 200 cycles at 2 A g^−1^) when used as a freestanding anode for LIBs. In addition, Guo et al. proposed a bimetallic Ni_x_Co_y_-silicate@CNTs flexible thin-film electrode by using CNTs to bridge and interconnect Ni_x_Co_y_-silicate nanosheets, which overcame the problem of the low number of redox pairs and poor conductivity of the conventional silicate material. [30] This electrode structure provided suitable channels for the rapid transport of lithium ions and electrons, ensuring a high flexibility of electrode materials.

Similarly, CNTs with high electrical conductivity can play an equally important role in forming cathodes with active materials for LIBs with excellent electrochemical properties. Li et al. assembled a full battery with lithium iron phosphate (LFP)/CNTs/CNFs as the cathode and lithium titanate (LTO)/CNTs/CNFs as the anode [4]. CNTs enhanced the conductivity of the electrodes by forming a 3D conductive network, while a dual conductive network of electrons and stress release was formed in the entanglement of CNFs with the active material, which provided a strong guarantee for the maintenance of the performance of the flexible electrode. In addition, the use of directional freezing-assisted 3DP was able to produce well-oriented directional pore structures, which significantly shortened the ion transfer path in the vertical direction. These measures improved the electrochemical performance of the electrodes. The assembled full cell showed a high energy density (15.2 mWh cm^−2^) and a high power density (75.9 mWh cm^−2^), which provided a feasible preparation solution of high energy density flexible LIBs with dynamically stabilized power output.

The metal foil current collector is a very heavy electrochemically inert component that does not contribute to the capacity and reduces the overall energy density of the battery [31]. Moreover, metal foils have smooth surfaces with low yield strains [32] and weak adhesion to the electrode materials during mechanical bending, leading to a fast capacity attenuation and poor rate performance [33]. Studies have shown that lighter carbon nanotube current collectors can improve the mechanical stability, adhesion strength and wettability of active materials, and their interconnection networks can maintain high conductivity when bent. To fabricate a highly conductive MWCNT coated paper, Ventrapragada et al. [34] developed an additive-free and roll-to-roll spraying process coating CNTs onto commercial cellulose-based paper. Subsequently, MWCNT coated paper was tested as current collectors for LFP LIBs. As a result, the area capacity of the LFP coated paper carbon nanotube cathode was increased by about 17% compared to that of the aluminum-based LFP cathode, and the assembled LIBs exhibited a high energy density of 460 Wh kg^−1^ at a power density of 250 W kg^−1^. Sharma et al. prepared a novel polar current collector by depositing a thin layer of carbon nanotube and polymer paste on aligned CFs (CF-CNT-P) [31]. The CNTs provided a uniform current density, while the carbon fibers (CFs) improved the electronic conductivity and mechanical strength of the CF-CNT-P (Figure 2c) and the polymer enhanced the adhesion of the active materials coating to the current collectors. When compared to deposition on conventional Al foils current collectors, the constructed NCM622 cells using CF-CNT-P composites as the cathode current collectors exhibited a lower charge transfer resistance, higher rate performance, and higher cycling stability.

#### 2.1.2. Graphene-Based Electrodes for Flexible LIBs

Graphene has a variety of industrial applications for flexible displays, electronics, energy storage systems, etc. [35]. The fabrication of graphene-based electrodes to build flexible batteries has received much attention, including metal-ion batteries, lithium-sulfur batteries, and metal-air batteries [36]. Carbon atoms within graphene materials are bonded and tightly stacked in a sp2 hybridized manner to form a single-atom layer structure with a special hexagonal crystal shape [35,37]. The honeycomb structure of 2D graphene is a promising substrate for flexible electrodes, because of its light weight, excellent electrical conductivity, and superior mechanical stability [36,38].

As mentioned earlier, TMOs are promising anode materials for LIBs. However, due to the low conductivity and the large volumetric expansion in charge/discharge progresses, the TMOs-based electrodes tend to exhibit fast capacity attenuations in long-term cycling. Combining TMOs nanostructures with carbonaceous materials is an effective method to improve electrical conductivity and buffer the volume change. Ma et al. successfully synthesized uniformly distributed TiO_2_ nanorods/reduced graphene oxide (rGO) composites (TNGC) (Figure 2d) by a simple sol-gel static self-assembly method and alkali heat treatment [39]. The nanorod structure of TiO_2_ can provide shortened diffusion distance for Li^+^ and the rGO network can greatly increase the electrical conductivity of the TNGC and prevent the overgrowth of TiO_2_. Zhao et al. formed a conductive network by growing uniformly small Fe_2_VO_4_ nanoparticles (FVO) on rGO (FVO/rGO) via solvent heat treatment and subsequent calcination [40]. The FVO/rGO composites anode exhibited a high initial capacity of 1013.7 mAh g^−1^ and maintained a stable capacity of 500 mAh g^−1^ after 300 cycles in LFP LIBs (Figure 2e). In addition, Nguyen et al. synthesized a few-layer NbSe_2_@graphene (FLNG) composite by wet ball milling (WBM) (wet ball milled NbSe_2_@graphene or WBMNG) (Figure 2f) [41]. The results for the WBMNG/Li half-cell showed a highly stable cyclic performance (~700 mAh g^−1^ at 1 A g^−1^ after 1000 cycles) and excellent rate performance (~76% capacity retention at 10 A g^−1^ compared to the capacity at 0.1 A g^−1^), suggesting that it can also serve as a new promising anode for LIBs due to its excellent electrochemical properties.

LiNi_x_Mn_y_Co_(1−x−y)_O_2_ cathodes with higher nickel content, such as LiNi_0.8_Mn_0.1_Co_0.1_O_2_ (NMC811), have high specific capacity but serious safety and cycle life issues [42]. Setyawati et al. added graphene to the NMC811 by simple solid-state mixing and subsequently assembled 18,650 cylindrical cells with commercial graphite anode [43]. As a result, the graphene-modified NMC cathode material showed a capacity retention of 95.83% at 1 C after 100 cycles, which was only 92.27% of the graphene-free NMC. Moreover, the highest temperature recorded during charging and discharging processes was only 46.52 °C at 5 C. Organic cathode materials have received increasing attention in LIBs due to their abundance, environmental friendliness, high specific capacity, low cost, and flexibility. However, their poor electrochemical performance due to their inherent low conductivity and high solubility in polar organic electrolytes has hindered their application. Sui et al. loaded 4,8-dihydrobenzo (1,2-b:4,5-b’) dithiophene-4,8-dione (BDT) into the nanopores of 3D graphene to prepare BDT/3D graphene composites by a simple impregnation method [44]. Due to the excellent domain-limiting effect of the nanopores and the strong π-π interactions with BDT, 3D graphene avoided the dissolution of BDT in the electrolyte. Additionally, graphene networks can provide a highly conductive framework with a large number of interconnected pores. Thus, obtained BDT/3D graphene composites showed greatly improved a rate performance of 100 mAh g^−1^ at 4 C and a cycling stability with capacity retention of 80% at 0.5 C over 200 cycles (Figure 2g).

The inherent monolayer structure of graphene enables the easy construction of 2D graphene paper, which exhibits excellent mechanical flexibility and superior electrical conductivity, and can be repeatedly bent or rolled up without any breakage. Graphene paper has been used as a flexible current collector to support various active materials such as LFP, MoO_2_, TiO_2_, and sulfur for flexible batteries [36]. Recently, Xu et al. dispersed rGO in water to form a colloidal suspension [45]. Subsequently, it was coated on a polyethylene terephthalate (PET) substrate and dried at 100 °C. Finally, graphene foil (GF) with high conductivity (~5800 S cm^−1^) and low mass density (1.80 g cm^−3^) was prepared after annealing at ultra-high temperature (2800 °C), which exhibited significant resistance to anode corrosion and achieved high energy density compared to conventional Al foil current collectors.

#### 2.1.3. Other Carbon-Based Electrodes

In addition to CNTs and graphene, flexible carbon-based materials used as flexible lithium batteries also include CF materials, such as CNFs, carbon paper, CF, etc., which are flexible while also having high electrical conductivity and high mechanical strength and are considered to be very promising flexible electrode materials.

**Figure 3:**
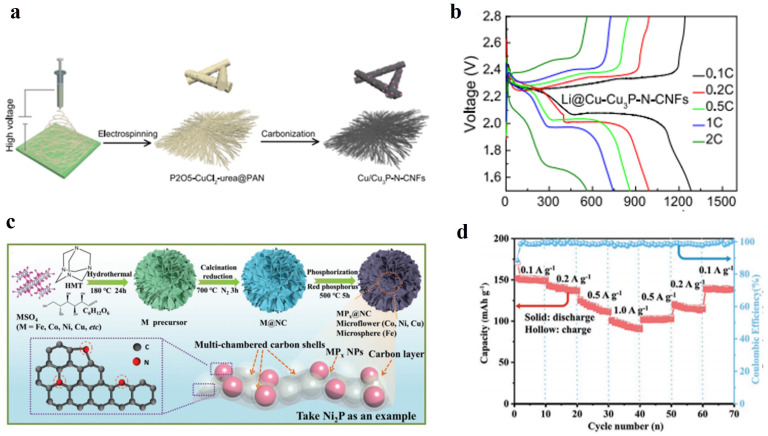
Other flexible carbon-based materials. (**a**) The synthesis procedure of Cu/Cu_3_P-N-CNFs current collector and (**b**) charge and discharge curves of Li-S battery assembled with Cu_3_P-N-CNFs anode. (**c**) The synthesis procedure of the MP_x_@NC composite materials and (**d**) rate performance and coulombic efficiency of Ni_2_P@NC//LFP full cell. Reprinted with permission from Reference [51].

With the advantages of high electrical conductivity, good flexibility, low cost, and large-scale preparation, CNFs are regarded as flexible electrode materials with broad application prospects. Although surface functionalization and atomic doping can improve the electrochemical performance of pure CNFs electrodes, the specific capacity and capacitance of CNFs are still unable to meet the growing requirements of the flexible electrochemical energy storage (EES) [46]. Therefore, CNFs also need to be combined with other electrochemically active materials to obtain the ideal composite electrodes with high flexibility and high energy density. Huang et al. successfully prepared three-dimensional monolithic flexible composite anodes consisting of CF coated with N-doped porous exfoliated CF, NiO, and carbon quantum dots (CD) (CF/ECF/NiO/CD) [47]. Subsequently, a 45.0 cm^2^ soft-pack full battery using commercial NCM cathode was designed and delivered a high areal capacity (41.33 mAh cm^−2^), volumetric capacity (413.3 mAh cm^−3^), and mass capacity (134.5 mAh g^−1^) at high current density of 5 mA. The multi-shell (CF/ECF/NiO/CD) exhibits excellent lithium storage performance as a negative electrode material for LIBs half-cells. Savignac et al. utilized CF waste from the aerospace industry to fabricate electrodes [48]. They first surface treated the recycled CFs and then dispersed them homogeneously in an active material consisting of LiFePO_4_ and 3,4-ethylenedioxythiophene (EDOT) using an easily scalable one-pot process. The resulting LiFePO_4_-based freestanding electrodes (FSEs) showed a total electrode capacity of 141 mAh g^−1^ and an energy density of 468 Wh kg^−1^ due to the absence of current collectors and avoided high costs at the same time. CNFs can also be used to construct flexible current collectors. For example, Xiang et al. designed and fabricated a 3D current collector consisting of nitrogen-doped porous CNFs decorated by copper-based Cu/Cu_3_P heterostructure (Cu/Cu_3_P-N-CNFs) (Figure 3a) for stabilizing lithium-metal anode [49]. The Li-S full battery equipped with Li@Cu/Cu_3_P-N-CNFs anode achieved a long lifetime and excellent electrochemical performance at a low N/P (about 2:1) and a high sulfur loading capacity (4.1 mg cm^−2^) (Figure 3b). Chen et al. employed ion exchange to synthesize carbon-coated NiO nanosheet arrays on the CF surface of a flexible 3D porous CC [50]. The effective carbon coating can not only enhance the electron transfer kinetics and structural integrity of the electrode, but also buffer the repeated volume change of NiO during cycling progresses. Even at a high mass loading of 4 mg cm^−2^, C@IENiOCC still possessed an excellent area capacity of 3.08 mAh cm^−2^ and a high initial coulombic efficiency of 91.2%. In addition, Cui et al. encapsulated monodisperse transition metal phosphides (MP_x_) into flexible nitrogen-doped carbon multi-chambers (MPx@NC, M = Ni, Fe, Co, and Cu, etc.) with pre-preserved voids as the anode for LIBs (Figure 3c), which can achieve high electronic conductivity and fast reaction kinetics, shorten the lithium transport path, and buffer the large volume change [51]. The full battery assembled with Ni_2_P@NC anode and commercial LiFePO_4_ cathode showed a high reversible capacity of 150.1 mAh g^−1^ and a high capacity retention of 87.4% after 100 cycles at 0.1 A g^−1^ (Figure 3d).

In order to meet the requirements of flexibility and energy density of LIBs, flexible carbon-based materials need to be combined with active materials to form an integrated electrode. Table 2 summarizes and compares the synthesis methods, flexibility, conductivity, and electrochemical properties of different electrode materials. Different carbon-based materials have their own advantages and disadvantages; for example, CNFs can provide enough space to support the active substance, alleviate the volume expansion of the active material during cycling, and can also promote electron transfer. However, its functional content is low, which makes the binding force between the active substance and the nano-CF weak. Therefore, morphology engineering can improve the lithium storage capacity and cycle stability of carbon-based composites by constructing a micron-sized 3D structure composed of nanostructured units. Therefore, in the future, it is necessary to conduct in-depth research on the morphology, interface structure, loading of active substances, and adaptation between active substances and carbon-based materials to ensure that the composite electrode has both high flexibility and high energy density.

### 2.2. Flexible Lithium-Based Anodes

Despite the significant advantages of LMBs in terms of energy density, the use of lithium metal for flexible lithium anodes faces some obstacles: (1) the random growth of lithium dendrites and large volume changes during charging/discharging, which cause the formation of unstable solid electrolyte interface resulting in a reduction of CE, even in the short-circuit of the battery; (2) lithium metals’ own mechanical flexibility is insufficient, and the electrode material is prone to cracking under stress, exacerbating the uneven deposition of lithium and ultimately reducing the electrochemical performance of the electrode [5,52]. In order to develop flexible lithium metal anodes with high mechanical stability and electrochemical activity, people have begun to study the flexible lithium matrix and flexible interface.

#### 2.2.1. Flexible Lithium Hosts

**Figure 4:**
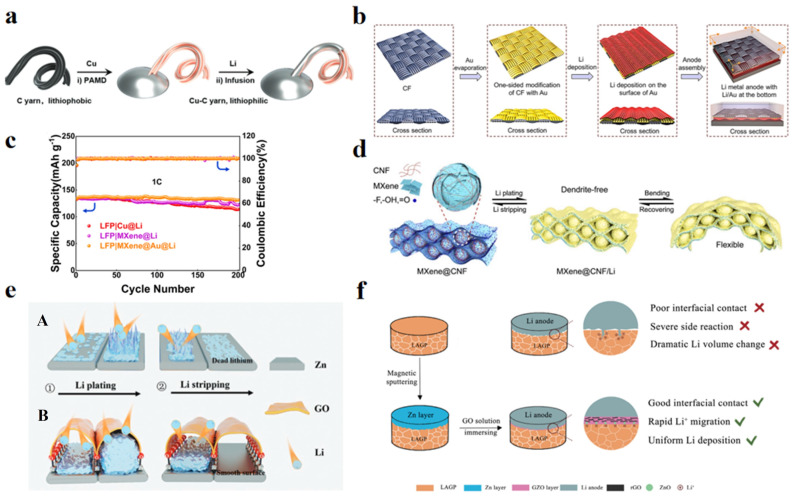
Flexible lithium anode materials and their properties. (**a**) The preparation procedure of LMCY. Reprinted with permission from Reference [53]. (**b**) The synthesis process of Li/AuCF anode. Reprinted with permission from Reference [56]. (**c**) The cycling performance of lithium batteries at 1C of LFP|Cu@Li, LFP|MXene@Li and LFP|MXene@Au@Li. Reprinted with permission from Reference [57]. (**d**) Schematic illustration of the process of lithium plating and stripping on MXene@CNF. Reprinted with permission from Reference [58]. (**e**) Schematic illustration of lithium metal plating/stripping process on different substrates. (A) Exposed zinc nanosheets and (B) An interface layer with tent shaped nanocavities anchored by Zn-O-C bonds. Reprinted with permission from Reference [59]. (**f**) Schematic illustration of LAGP/Li interface modified with rGO/ZnO (GZO). Reprinted with permission from Reference [61].

The realization of flexible lithium hosts mainly starts from two aspects: the first is to directly improve the mechanical flexibility of lithium metal. For example, simply processing lithium metal anodes into lithium foils and wires can indeed provide a certain degree of flexibility when LMBs are subjected to slight mechanical deformation, but there are still problems such as dendrite formation, low yield strength, and poor fatigue resistance [53,54]. Gao et al. designed flexible and stable lithium-metal composite yarns (LMCY) by fast capillary filling of molten lithium into metallic carbon yarns that were used to fabricate high energy density and long-lasting linear LMBs (Figure 4a) [53]. As a result, LMCY displayed excellent electrochemical cycling stability, mechanical strength, flexibility, and durability. The foldable LFP||Li full battery obtained by matching LMCY with LFP had a volumetric energy density of more than 290 Wh L^−1^ and a capacity retention rate of more than 50% after 750 charge/discharge cycles.

The second is the development of composite lithium metal anode (CLMA) materials. Through the intervention of doping lithophilic components to regulate the lithium metal deposition process, uniform lithium deposition/stripping can be realized and the formation of lithium dendrites can be suppressed. At the same time, the composite of lithium metal and flexible 3D porous material forms a high specific surface area and porous structure, which can reduce the local current density, buffer the volume change, and improve the electrode flexibility. For example, lithium metal anodes based on 3D carbon-based fabrics present a promising strategy for achieving high-performance flexible lithium metal batteries (LMBs) [55]. Li et al. utilized a lithophilic Au layer with a simple modification to the bottom of the CFs (Figure 4b) to direct the directional deposition of Li onto the bottom of the CFs to achieve a favorable lithium-free anode/diaphragm interface, while the porous skeleton in the upper part of the CFs provides a large space to buffer the volume expansion of lithium metal [56]. The cycle life of the resulting composite lithium metal anode was doubled compared to conventional top-deposited lithium metal. Qian et al. designed a 3D flexible MXene@Au host via depositing an ultrafine and robust Au layer on MXene paper by ion sputtering [57]. The Au layer acted as a lithium nucleating agent to ensure uniform lithium deposition and inhibit the growth of lithium dendrites. The results showed that the LMBs with LFP cathode had a superior capacity retention rate of 98.47% even after 200 cycles (Figure 4c). Wang et al. synthesized freestanding, lightweight, and flexible MXene membranes through inducing the assembly of MXene dispersions containing trace amounts of CNFs via rotary vaporization technique (Figure 4d) [58]. The interlocking between MXene sheets and microspheres significantly enhanced the toughness of the MXene film and prevented the restacking of MXene. In addition, the MXene nanosheets, with abundant polar functional groups, exhibited good lithium affinity, enabling uniform Li deposition, while the highly conductive network facilitated rapid charge transfer. The composite lithium film was extremely robust, capable of bending significantly and returning to its original state without cracking. Benefiting from these advantages, the flexible mixed lithium anode, when paired with a flexible LiFePO_4_/cellulose nanofiber (LFP@CNF) cathode, enabled the construction of fully flexible LMBs with high specific capacity and excellent stability.

#### 2.2.2. Flexible Interfaces

The unstable interface (inhomogeneous interfacial composition and morphology) formed between the lithium metal anode and electrolyte of LMBs leads to an inhomogeneous surface current distribution. Li^+^ tends to deposit at high current density sites and induces dendrite growth. Due to its high reactivity, Li metal spontaneously reacts with organic components in the electrolyte to form brittle solid electrolyte interphase (SEI) on the Li surface. The cracks generated by SEI rupture further and can lead to increased current density and dendrite problems due to its inability to withstand large volume changes during Li deposition/stripping. With the crushing and fracturing of the dendrites, “dead” lithium is formed, which hinders the rapid transport of Li^+^ and further increases the interfacial impedance. This ultimately leads to reduced CE and limited cycle life of LMBs. Therefore, enabling ideal high performance flexible LMBs demands regulating the uniform deposition/stripping of lithium and suppressing the growth of dendrites. It is a very important strategy to design and fabricate highly stable and flexible electrode/electrolyte interfaces.

Several strategies have been used to design and construct electrochemically stable and mechanically flexible SEI to regulate uniform Li deposition and achieve long cycle life. In situ modification is an effective strategy to build stable interfaces by changing electrolyte components such as additives, salts, and solvents so as to form a homogeneous passivated SEI on the lithium metal surface. Ci et al. utilized the facile reduction reaction between Zn and GO solution sputtered onto the Li_1.5_Al_0.5_Ge_0.5_(PO_4_)_3_ (LAGP) surface at room temperature to in situ prepare a rGO/ZnO (GZO) flexible film to modify the LAGP/Li interface (Figure 4e). The flexible layered rGO film improves the interfacial contact, ZnO homogenizes the Li+ flux, and the GZO flexible film prevents side reactions between the lithium metal anode and LAGP. Consequently, the LFP/Li full cell with GZO@LAGP exhibits a high capacity retention ratio of 93.8% after 100 cycles under 0.5 C at 25 ℃ [59].

Another common strategy is the ectopic interfacial modification, which minimizes the contact between lithium metal and electrolyte by constructing a passivation layer on the surface of the lithium metal anode, reducing the side reactions. Li et al. constructed a double-layer artificial SEI on metallic lithium based on a spray quenching method. The upper layer consisted of a flexible organic component, while the lower layer contained a large amount of LiF and lithophilic zinc nanoparticles. The zinc nanoparticles regulated the Li^+^ flux through lithophilic sites. The fluorinated interfacial layer exhibited significant stability, effectively protecting lithium from the corrosive electrolyte and suppressing dendrite growth [60]. As a result, the improved lithium electrode was stable for more than 400 cycles in a symmetric cell at a current density of 3 mA cm^−2^. Zhang et al. designed a unique interface with flexible tent-like nano-cavities using a simple self-assembly method to achieve a reversible lithium metal anode and reduce electrolyte interference (Figure 4f). The Zn-O-C bonds exhibited excellent affinity for lithium metal and acted as lithophilic sites to guide uniform nucleation within the nano-cavities. Meanwhile, the flexible tent-like interface provided greater space to accommodate lithium deposition and restricted the plating/stripping process within the nano-cavities, facilitating dendrite-free lithium deposition. The flexible rGO layer enhanced the mechanical flexibility of the tent-like interface. As a result, the full cell assembled with LFP cathode exhibited excellent long-term cycling stability and rate capability, maintaining a high capacity of 94.6% after 3000 cycles at 5 C [61].

### 2.3. Flexible Electrolyte Materials

SSE can generally be classified into three major categories: ISE (crystals, glass, ceramics, etc.), SPE, and CSE [62]. Compared to liquid electrolytes, SSE can simultaneously reduce leakage risks and suppress lithium dendrite formation. They are widely applied in flexible and wearable LMBs to replace traditional liquid electrolytes [16]. In practical applications, the use of SSE requires addressing several key issues, including insufficient flexibility, low ionic conductivity, and the interface resistance generated when the electrolyte-electrode interface bends.

#### 2.3.1. Inorganic Solid-State Electrolyte

ISE exhibits excellent mechanical properties, high ionic conductivity (10^−6^–10^−4^ s m^−1^) at room temperature, wide electrochemical windows (>6 V), and high chemical stability towards lithium metal. Due to its outstanding mechanical properties and high elastic modulus, it can suppress the cracking caused by lithium dendrite growth. However, this makes the toughness of ISE poor, increasing the practical application difficulty of flexible electronic products [52]. Recently, some studies have provided several effective strategies for designing flexible SSE.

Using electrospinning technology to prepare inorganic SSE can result in uniform distribution of electrolyte precursors within hybrid nanofibers, enabling the solid-state electrolyte to possess a certain degree of flexibility. Wang et al. prepared Li_6.4_La_3_Zr_2_Al_0.2_O_12_ (LLZO) templates through electrospinning and template methods and combined them with PVDF based solid electrolyte containing high-concentration lithium bis(trifluoromethanesulfonyl)imide (LiTFSI) and LLZO with a 3D framework, achieving superior performance in the composite solid electrolyte. Experiments show that the CSE with a 3D backbone has a breaking stress of 1.4 MPa and an elongation of 53% [63]. Furthermore, most inorganic SSE possess rigidity and are susceptible to mechanical strain. Combining them with flexible polymers can effectively suppress their brittleness and enhance mechanical strength. Sreejith et al. proposed to combine a garnet-structured fast ion conductor (Li_6.28_Al_0.24_La_3_Zr_2_O_12_) with PVDF polymer (in a ratio of 8:2) through a roll-to-roll process to form a standalone flexible ceramic-polymer electrolyte [64]. Additionally, we can utilize fabric as a template to construct inorganic SSE with flexible structures. Pan et al. utilized silk as a template to fabricate layered-structure garnet LLZO ceramic fabric composite solid electrolyte (LLZO-CF-CSE). This flexible LLZO-CF-CSE possesses the morphology and structure of original silk fabric, thereby offering a 3D rapid ion conduction continuous pathway [65]. Although ISEs have enhanced mechanical toughness through technical means, they still face significant challenges in achieving the commercialization of flexible electronic products due to their pronounced mechanical rigidity.

#### 2.3.2. Solid Polymer Electrolytes

SPE have high elasticity and high stability towards lithium metal anodes. They are easily prepared into a fibrous or sheet structure and thus can be used as an ideal electrolyte material for flexible lithium batteries. The easy processability of organic polymers endows them with adjustable mechanical and electrochemical properties, including excellent flexibility and mechanical stability, wide compatibility, and high ionic conductivity, owing to their structural diversity and modifiability.

**Figure 5:**
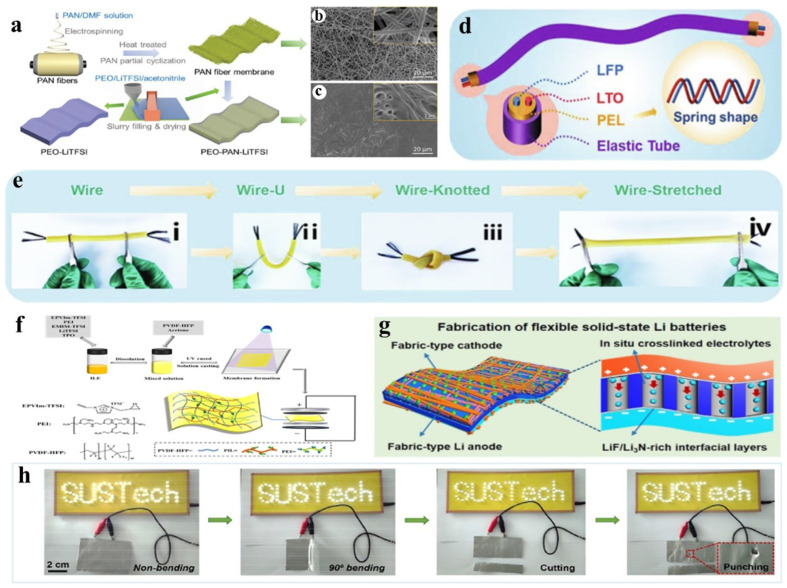
(**a**) Preparation of flowcharts of PEO-LiTFSI and PEO-PAN-LiTFSI. (**b**,**c**) SEM images of (**b**) the PAN fiber membrane and (**c**) PEO-PAN-LiTFSI. Reprinted with permission from Reference [66]. (**d**) Diagram of wire-shaped cell. (**e**) (i)–(iv) Photographs of wire-shaped cells in different deformations. Reprinted with permission from Reference [67]. (**f**) Schematic illustration for the synthesis of DN-Ionogel. (**g**) Schematic diagram of a flexible all-solid-state lithium battery and a hybrid interface composition during cycling. (**h**) The upholstery unit provides a display that powers the optical image of a large LED screen in a variety of conditions such as raw, bent, cut, and stamped. Reprinted with permission from Reference [69].

Currently, the modification strategies of SPE used in flexible batteries include crosslinking, copolymerization, and blending methods. Crosslinking is a modification method in which polymer chains are connected through chemical bonds to form a three-dimensional network structure. This approach can significantly enhance the mechanical strength and thermal stability of the polymer while disrupting the ordered structure of the polymer chains. As a result, the amorphous regions are considerably expanded, leading to an increase in ionic conductivity. Sheng et al. designed a crosslinked copolymer membrane electrolyte of polyethylene oxide-polyacrylonitrile (PEO-PAN) incorporating polyacrylonitrile (PAN) fiber fillers. To construct a PEO-PAN crosslinked membrane with PAN fiber filler (Figure 5a), PAN fibers, which exhibit a uniform diameter distribution within the range of 400–500 nm, can be used (Figure 5b). After the addition of PEO and lithium salt, the spaces between PAN fibers are uniformly filled (Figure 5c). Due to its high flexibility and strong binding ability, this electrolyte maintains good interfacial contact with both the cathode and anode during bending, ensuring stable electrochemical performance of flexible lithium batteries [66]. Liu et al. developed a resilient butyl polyacrylate-based SPE(PEL) using butyl polyacrylate crosslinked polyethylene glycol (PEGDA) and 1-ethyl-3-methylimidazoline bis(trifluoromethylsulfonyl)imide salt (EMIMTFSI). Figure 5d demonstrates the incorporation of a PEL electrolyte into cylindrical batteries, with electrodes arranged in a spring-like configuration to impart stretchability. In U-bending, knotting, and tensile conditions (Figure 5e), although batteries undergo various deformation processes, their corresponding impedance values do not change significantly, demonstrating the suitability and feasibility of flexible PEL electrolyte shapes [67]. Liang et al. synthesized double-network-supported polymeric ionic liquid-based ionic gel electrolytes (DN- Ionic gel) via a simple one-step crosslinking method (Figure 5f). This dense crosslinked structure not only securely locks EMIMTFSI without leakage but also exhibits excellent flexibility [68]. Wen et al. synthesized an ultra-thin cross-linked SPE for flexible lithium batteries by one-step in-situ crosslinked polymerization of 1, 3-dioxolane and trimethylolpropane triglycidyl ether within a lithium nitrate-containing mesoporous polymer (LP) matrix, which improved the mechanical strength of SPE by forming a high-modulus and high-conductivity Li_3_N/LiF during cycling (Figure 5g). Power supply for the high-voltage All-Solid-State Lithium Battery (ASSLB) light-emitting diode display maintains screen brightness under bending, cutting, and punching conditions (Figure 5h). Compared to liquid and gel polymer electrolytes, integrated ASSLBs exhibit high capacity retention (>90%), minimal voltage fluctuation (<50 mV), and enhanced safety during 2000 bending cycles (bending radius: 5 mm) [69].

Condensation polymerization is another method to prepare flexible SPE. Wu et al. synthesized self-healing solid polymer electrolytes (SHSPEs) with rigid-flexible frameworks and high ionic conductivity via a simple condensation reaction. The cross-linked poly (ethylene glycol)-based thermoplastic polyurethane (TPU) network, featuring dynamic intermolecular/intramolecular hydrogen bonding interactions, endows the electrolyte with a rigid-flexible coexistent structure, rapid self-healing capability, and outstanding electrochemical properties [70].

#### 2.3.3. Composite Solid-State Electrolyte

CSEs combine ISE and SPE and inherit the advantages of both in terms of performance. In recent years, due to its ability to enhance electrochemical, thermal, and mechanical properties, CSE has been widely studied. In flexible lithium batteries, CSE can still maintain the inherent excellent flexibility and processability of polymer components, making it a highly promising solid electrolyte material.

**Figure 6:**
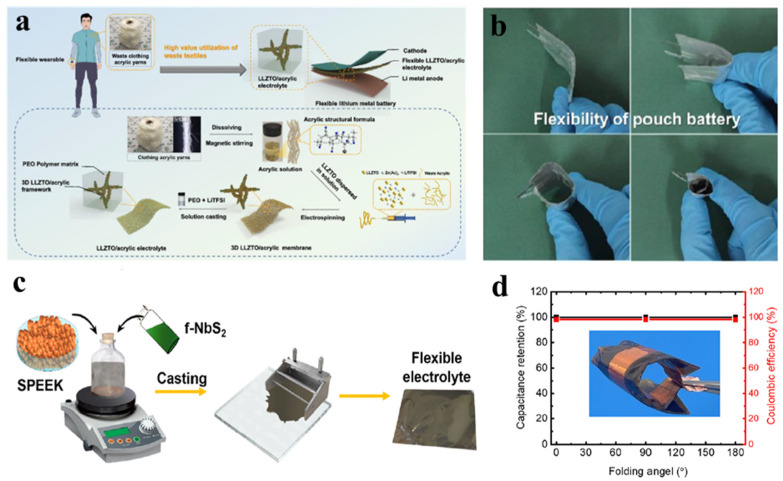
(**a**) Above: Flowchart of converting discarded clothing acrylic into a flexible wearable device. Bottom: Schematic diagram of the preparation of composite electrolytes from waste acrylic yarn. (**b**) Demonstration of the flexibility of pouch cells. (**c**) Flow diagram for the preparation of a solid electrolyte by incorporating f-NbS_2_ nanosheets into a SPEEK matrix. (**d**) Capacitance retention and CE of FSSSC under different folding conditions. Reprinted with permission from Reference [6].

Pomegranate-type Li_7_La_3_Zr_2_O_12_ (LLZO) solid electrolyte, known for its high ionic conductivity at room temperature (10^−3^–10^−4^ S cm^−1^) and stability towards lithium metal, has been extensively researched [71]. Zhang et al. mixed oxide garnet ceramic Li_6.4_La_3_Zr_1.4_Ta_0.6_O_12_ (LLZTO) nanoparticles with acrylic acid and electrospinning technology to prepare a flexible solid electrolyte with a nanofiber framework (Figure 6a). The all-solid-state pouch battery prepared by using the electrolyte can be bent at any angle (Figure 6b), and the open-circuit voltage of the battery is maintained at about 3.4 V in the flat or bent state of the battery [72]. Besides introducing acrylic acid, poly (ethylene carbonate) can also be utilized as a polymer to incorporate LLZTO material. This CSE exhibits excellent comprehensive performance at 20 °C: high ionic conductivity (5.2104 S cm^−1^), a wide electrochemical window (4.6 V), a high ionic transference number (0.75), and satisfactory mechanical strength (6.8 MPa) [73]. In addition, the use of doping, hybridization, and additives is an effective strategy for constructing flexible battery composite solid electrolytes. Bagheri et al. prepared flexible SSE by composite fabrication of metal-organic framework-5 transition metal disulfide, namely liquid-phase exfoliated functionalized niobium disulfide (f-NbS_2_) nanoflakes, with a sulfonated poly (ether ether ketone) (SPEEK) polymeric matrix (Figure 6c). Doped NbS_2_ flakes can facilitate the interaction between introduced -SO_3_H groups and the SPEEK matrix via hydrogen bonding, maintaining good capacitance in flexible batteries folded at 0°, 90°, and 180° (Figure 6d), endowing the composite electrolyte with excellent mechanical and electrochemical properties [6]. Hybridization of solid-state organic-inorganic components can overcome the drawbacks of low ion conductivity in inorganic materials and narrow electrochemical stability windows in organic materials. Baek et al. introduced lithium phosphate (Li_3_PO_4_) as the support and safety medium, and proposed to coat Li_3_PO_4_ with solidified 1-ethyl-3-methylimidazolidinium bis(trifluoromethylsulfonyl)imide lithium (Li-EMI-TFSI) as a hybrid electrolyte, which has the advantages of long-term operation, safety, and flexibility [74]. Adding functional inorganic additives is an effective way to improve its performance, including enhancing ion conductivity, achieving dendrite inhibition, and improving the safety and stability of flexible batteries [75].

The research on the key flexible materials for flexible batteries is summarized as follows. Carbon-based materials are one of the important materials for the construction of flexible current collectors and pole pieces. Among them, carbon nanotubes and graphene have the advantages of good conductivity and strong plasticity and are widely used in the research of flexible materials, and have good development prospects. At present, the research on flexible lithium base is mainly focused on solving the problem of flexible lithium host and interface contact. The main modification strategy used is to structurally construct the flexible material with lithium or to add additives and salts to enable the lithium to be evenly deposited. However, these modification strategies have certain limitations, so there is still some room for research. For flexible electrolytes, composite electrolytes can meet the conductivity of traditional electrolytes while also being mechanically flexible, so they have better development potential in flexible battery components.

## 3. Typical Flexible Battery Fabrication Process

During the preparation process of flexible batteries, selecting appropriate techniques is crucial for the integration of various flexible components. In traditional flexible battery fabrication methods, ensuring the stability of quality at contact interfaces or junctions is challenging. Particularly during bending, there is a risk of poor contact or insufficiently secure connections, which may lead to malfunctions [17]. With the development of advanced process technologies, the preparation technology of flexible batteries has been improved. The following discusses several typical processes for preparing flexible materials.

### 3.1. 3D Printing

3DP is a form of rapid prototyping technology that utilizes materials such as powdered metals or plastics that can be bonded together to construct objects layer by layer. It has the unique advantage of producing energy storage devices with outstanding characteristics such as microscale size and aesthetic diversity, making it an important technology widely used in miniaturization and customization of electronic products [76].

**Figure 7:**
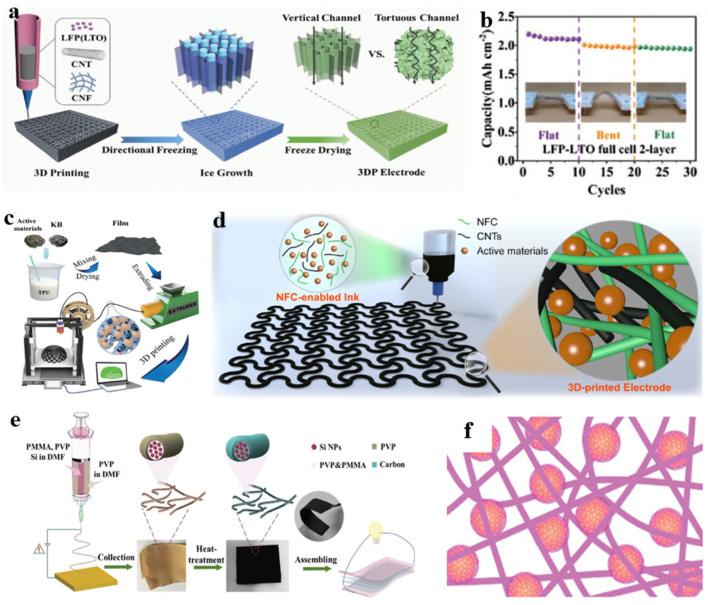
(**a**) Flow diagram of flexible electrode preparation by 3DP technology. (**b**) Cycling performance of the whole battery in both flat and curved states. Reprinted with permission from Reference [4]. (**c**) Schematic illustration of the fabrication process of the 3D-printed TPU-based electrodes. Reprinted with permission from Reference [78]. (**d**) Composition and morphological characteristics of retractable cell components, and advantages of retractable cells. Reprinted with permission from Reference [79]. (**e**) Schematic diagram of the preparation of flexible Si@C NF using electrospinning technique. (**f**) Structural diagram of a flexible fiber paper electrode before carbonization. Reprinted with permission from Reference [8].

In the process of preparing flexible batteries, utilizing 3DP can integrate the target device into a model, effectively optimizing interface contact and ensuring close adherence of flexible devices, thereby enhancing the mechanical resilience and electrochemical performance of the battery. Li et al. proposed using directional freezing to assist 3DP in constructing flexible and compressible LIBs (Figure 7a), forming an electronic/stress dual-conductive network and introducing directional pore structures to ensure rapid electron transfer and stress release throughout the printed electrodes. The area capacity of the bilayered LFP/lithium titanium oxide (LTO) full battery reaches a flat state of 92.9% under bending, demonstrating excellent cycling stability (Figure 7b) [4]. Using 3DP technology to prepare the LFP/LTO electrode, Bao et al. tested the electrochemical performance and mechanical durability of the electrode under a wide range of and repeated instances of stretching and demonstrated the great potential of this printed electrode in realizing portable, wearable, stretchable, and flexible energy storage devices [7]. Additionally, research has shown that using reduced rGO-based printing ink to prepare flexible spinel-structured lithium titanate (Li_4_Ti_5_O_12_)/graphene fiber electrodes can establish a 3D conductive network within the fiber electrode, enhancing electronic conductivity and ion migration rate, thereby exhibiting excellent mechanical properties (35% elongation at break) [77].

Continuously optimizing the use of active materials based on 3DP can significantly enhance the flexibility of flexible battery materials. Hu et al. first proposed that TPU was developed as the host thermoplastic polymer for 3DP electrode filaments (Figure 7c). The TPU-LFP cathode and TPU-LTO anode soft-pack batteries prepared exhibited the best cycling performance among all 3D-printed batteries [78]. Qian et al. utilized extrusion-based 3DP to mix electrode active materials with nanofibrillated cellulose (NFC) to prepare serpentine-patterned stretchable electrodes and separators (Figure 7d). Ink based on NFC (Near Field Communication) possesses characteristics of high viscosity and shear deformation, enhancing the bonding between NFC elements and between NFC and active materials. Consequently, composite materials exhibit favorable mechanical properties. After 50 stretching cycles, the resistance of the electrode at 50% elongation increased by only 3% [79]. 

### 3.2. Electrospinning Technology

Electrospinning technology is a relatively simple and economically viable method for the preparation of flexible electrode technology. Through this technique, materials with high porosity can be prepared, enhancing wettability between the material and electrolyte, enlarging the contact area and thus favorably impacting the electrochemical properties of the components [80]. Carbon nanofiber materials prepared through precursor by electrospinning possess excellent flexibility, conductivity, high surface area, and manipulable structure, and are thus widely employed in the fabrication process of flexible lithium-ion battery components [81]. Combining elemental silicon, silicon-containing compounds, and metal compounds within CNFs can avoid issues such as poor adhesion due to the utilization of binders and the aggregation of nanomaterials.

The theoretical capacity of silicon is 10 times higher than that of graphite, making it a promising high-energy anode material for LIBs. However, due to the low loading of silicon on the heavy metal current collector and the significant volume change during the cycle, the overall electrode capacity of the silicon anode is still relatively low. Zeng et al. utilized electrospinning technology to fabricate flexible silicon/carbon composite nanofibers (Si@C NFs) with a core-shell structure (Figure 7e). The core-shell structure can not only act as a buffer to alleviate the adverse effects caused by the volume expansion of silicon nanoparticles (Si NPs) and avoid the direct explosion of Si NPs, but can also provide an efficient way to transport electrons and ions [82]. Similarly, PAN fibers produced by electrospinning combining electrically sprayed nano silicon–PAN clusters and subsequently carbonized to synthesize flexible electrodes. Figure 7f illustrates the structure of the flexible 3D Si/C fiber paper electrode before carbonization, with a CF network serving as a flexible and conductive matrix, with carbon-coated Si nanoclusters uniformly distributed. Strong adhesion is formed between the CFs and Si nanoclusters, enabling the battery to achieve a high silicon loading of 1.2 mg cm^−2^, with a total electrode capacity of approximately 1600 mAh g^−1^ [8]. Additionally, compressible nest-like sponge-like CNFs composite electrodes can be constructed using electrospinning technology with SiO_2_ nanoparticles. The unique sponge-like structure enables flexible batteries to possess exceptional compressibility and recovery capabilities under compression, while also exhibiting outstanding electrochemical performance [83].

Transition metal carbides (TMCs) can fill the interstitial gaps of anions, thereby enhancing the mechanical, chemical, and thermal performance of batteries. Lee et al. synthesized molybdenum carbide embedded in CNFs as the anode electrode (MoC/CNF) using amorphous molybdenum precursor and PAN as molybdenum source and carbon source, respectively, using a heating process in an N_2_ atmosphere. MoC/CNFs were employed directly as active electrodes, eliminating the need for binders, conductive agents, or current collectors [84]. Research also indicates that the crystallinity of Transition Metal Chalcogenides (TMC) also affects the performance of electrode materials. WS_2_/CNFs-500 nanofibers with the lowest crystallinity exhibit the best performance in LIBs due to their unique structure, where a large number of few-layer/single-layer WS2 nanosheets are uniformly dispersed within the framework of CNFs [85].

### 3.3. Coating

The coating process involves using a liquid slurry to attach active materials to a flexible substrate that serves as a support. After drying and other treatments, a composite material is obtained. In the preparation process of flexible battery materials, coating methods offer advantages such as low cost, ease of scalability, and good substrate selectivity.

**Figure 8:**
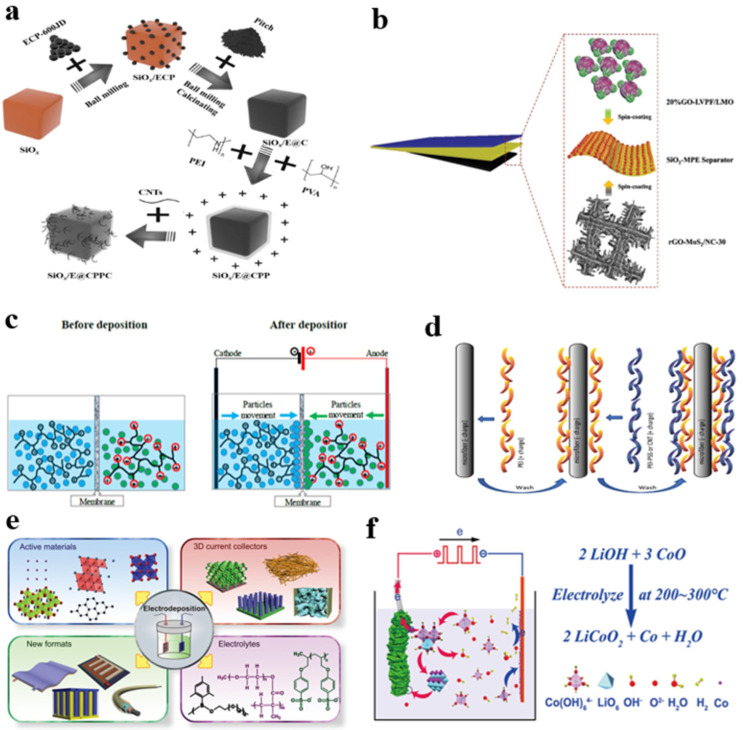
(**a**) Schematic chart of the fabrication for SiO_x_/E@CPPC. Reprinted with permission from Reference [86]. (**b**) Schematic diagram of a stacked thin-film cell configuration. Reprinted with permission from Reference [89]. (**c**) Schematic presentation of the simultaneous EPD process before and after application of electric field. Reprinted with permission from Reference [90]. (**d**) Schematic diagram of the preparation process of the flexible cathode current collector. Reprinted with permission from Reference [91]. (**e**) Overview diagram of electroplating each component of Li-based batteries. Reprinted with permission from Reference [92]. (**f**) Schematic illustration of electrodeposition process.

In the coating process, the active material, typically in powdered form, is usually mixed with a conductive agent and a binder, then poured into a solvent to form a slurry. Then, the slurry is thoroughly coated onto the substrate and dried to form a flexible electrode [17]. When preparing negative electrode materials using coating methods, flexible organic polymers and carbon materials are employed to encapsulate rigid silicon oxide (SiO_x_) (Figure 8a), thereby ensuring the structural integrity of SiO_x_ particles during volume changes [86]. Zhang et al. designed a self-supported diamond-shaped sulfur (D-sulfur)/single-walled carbon nanotube (SWCNT) composite cathode coated with poly(3,4-ethylenedioxythiophene) (PEDOT). The conductive PEDOT and SWCNTs effectively confine the dissolution of lithium polysulfides and successfully construct a flexible network [87].

Integrated flexible LIBs can also be directly fabricated by the Rod Coating Method [88]. Zhao et al. coated a composite anode(rGO-MoS_2_/NC-30) with a composite cathode (20%GO-LVPF/LMO) on both sides of a nano-silica-based modified PE separator (SiO_2_-MPE) by spin coating. (Figure 8b). This not only enables tight component coupling without relative displacement but also avoids the use of metal current collectors in battery configurations [89].

### 3.4. Electrophoretic Deposition

EPD is a technique that uses the movement of charged particles in an electric field for deposition. The particles are suspended in solution, move towards the electrode under the action of an electric field, and are deposited on its surface. The principle of the method is shown in Figure 8c, where negatively charged LTO particles and positively charged LFP particles in the electrolyzer are suspended in acetone solution and deposited on different matrices under the action of an external electric field. Since EPD is a solution-based technology, which allows the physical and chemical properties of the material to be preserved in the deposited thin film without being destroyed, it has received extensive attention in battery preparation [90].

At present, EPD can be used to prepare electrolytes and electrode assemblies with flexibility. Since most of the flexible current collectors of lithium batteries are fiber or braided, we need to embed the active electrode material into the current collector and ensure that the active material and the current collector have a good fit, so the EPD can be integrated with the flexible current collector without destroying the properties of the active electrode material [93]. Aliahmad et al. prepared a flexible cathode current collector by depositing oppositely charged polymers on microfibers (Figure 8d). This current collector has a high active material loading, and the assembled battery reaches 396 mAh g^−1^ and 300 mAh g^−1^ at 0.1 C and 1 C rates, respectively, with a capacity retention rate of more than 80% after 100 cycles [91]. In addition, the CC can be used as the negative plate in the electrophoresis tank using the EPD method, and LiFePO_4_ is dispersed in the electrophoresis solution. Under the action of the direct current field, LiFePO_4_ can be evenly loaded on the CC, thus forming a flexible integrated cathode. This current collector has a high active material load, and the assembled battery reaches 396 mAh g^−1^ and 300 mAh g^−1^ at 0.1 C and 1 C rates, respectively, and the capacity retention rate is more than 80% after 100 cycles [94]. At the same time, EPD can also be used to deposit ultra-thin CPE in situ on the LFP cathode. Since the EPD allows the OSE to be uniformly dispersed in the SPE, the contact between the CPE and the LFP cathode is improved [95].

### 3.5. Electrodeposition

Electrochemical deposition is a traditional technique that causes metal ions to undergo redox on the electrode surface and produce a thin deposition coating, mainly by applying an electric current to the electrode, as shown in Figure 8e. In the electrooxidation section, low-cost metal materials are mainly formed, and in the electroreduction section, high-value materials are mainly formed. In the process of material synthesis, the characteristics of the deposited layer are controlled by adjusting electrodeposition parameters such as current density and solution composition [92].

In contrast to EDP, redox reactions occur during electrochemical deposition. Therefore, it is mainly used for the deposition of metal and alloy materials, such as nickel, copper, gold, etc., and is often used as a technical method for the preparation of current collectors and electrodes for flexible batteries. For copper plating, a three-dimensional hollow flexible substrate such as CNT and CC is usually used as a carrier. The copper deposited by the electrochemical deposition method will have a special form, which can be better embedded in the three-dimensional flexible carbon material, so that the prepared lithium battery can maintain better electrochemical performance stability during deformation [96,97]. Alternatively, metal oxides can be deposited on flexible materials using electrochemical deposition methods. Cheng et al. grew Co_3_O_4_ nanosheet arrays (NSAs) on CC by electrodeposition. This hierarchical porous electrode structure not only inhibits the volume expansion of NSAs, but also facilitates electrolyte permeation and electron/ion transport [98]. By using the electrochemical deposition method, the mixture of V_2_O_5_ and graphene was deposited on CNT, and the current collector was prepared to release the tensile strain and compressive strain during the bending process and prevent the detachment of various active substances [91]. In addition to conventional electrochemical deposition methods, LiCoO_2_ films can be plated directly onto Al foil by performing low-temperature molten salt electrodeposition, resulting in a whole cell that can provide high-speed discharges of up to 20 C (Figure 8f) [99].

At present, most of the commonly used energy storage devices are based on rigid structures, so when the battery is bent or stretched, it is easy to cause failure or even explosion. This phenomenon is mainly due to the lack of sufficient elasticity of the metal current collector inside the electrode. Therefore, the design and fabrication of flexible current collectors are very important in the development of flexible batteries [100]. The role of the flexible current collector is mainly to support the conductivity of the active material and effectively maintain the flexibility of the electrode, which is usually based on CF, graphene, and soft metal current collectors. The preparation of flexible current collectors first requires the selection of suitable flexible substrate materials (with good mechanical flexibility and able to support flexible materials), and then the conductive materials need to be attached to flexible substrates; the production of these components often requires special processing technologies, and the above preparation process is also mentioned [101]. At present, the main current collectors and related battery module preparation technologies include 3DP [102], Electrodeposition [103,104], and EPD [105].

Table 3 summarizes the advantages and disadvantages of the five flexible materials and battery component preparation processes detailed above. The most prominent advantage of 3DP is the ability to create any geometry and the ability to build with high accuracy, but it is more expensive. Electrospinning technology is relatively easy to operate and has good uniformity, but it is less productive and more costly. The electrochemical deposition method is highly malleable and controllable, but the cost is high. In terms of battery performance and commercial mass production, 3DP technology is the most ideal for flexible battery manufacturing, and mass production can reduce the cost of battery production.

## 4. Various Types of Flexible Batteries

Flexible batteries are integrated with electrodes or electrolyte materials with flexible characteristics, which can meet the needs of general bending or folding. In addition to the research and preparation of flexible materials mentioned above, whether flexible batteries can function normally under significant deformation is an urgent issue that needs to be addressed. Therefore, the structural design of flexible batteries becomes particularly important. Currently, flexible batteries can be categorized based on their structural characteristics into spine-type batteries, foldable batteries, stretchable batteries, and cable-type batteries. The research progress by category is presented below, which is summarized in Table 4.

### 4.1. Flexible Battery with Joint-Inspired Structure

**Figure 9:**
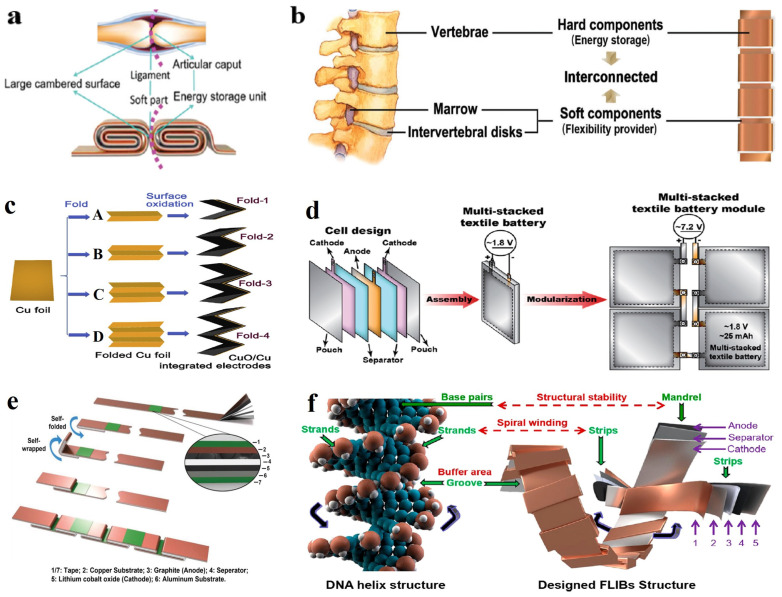
(**a**) Schematic diagram of a flexible structure battery for bone and joint bionics. Reprinted with permission from Reference [106]. (**b**) Design concept diagram of a battery with flexible structure based on animal vertebrae biomimicry. Reprinted with permission from Reference [107]. (**c**) Schematic diagram of the preparation of four CuO/Cu integrated folded electrodes. (**d**) Schematic diagram of a textile battery module. Reprinted with permission from Reference [114]. (**e**) Fabrication process of the zigzag-like foldable battery. Reprinted with permission from Reference [115]. (**f**) DNA helix structure and the helix-inspired battery design. Reprinted with permission from Reference [116].

It is still a challenge for flexible lithium batteries to achieve bending, folding, deformation stretching, and good mechanical properties at the same time. Therefore, people have begun to use the existing materials in nature to bionically apply them to the structural design of flexible batteries. For example, the human body makes various movements (bending and rotation) through complex neural regulation activities. The flexible battery designed by imitating the joint-ligament structure can provide the overall flexibility of the battery. The joint is like a battery that stores energy with a certain thickness and rigidity, and is connected by thin parts to achieve the flexible performance of the battery. (Figure 9a). It can also maintain stable cycling performance even after undergoing over 200,000 cycles of dynamic bending and 25,000 cycles of dynamic torsional deformation [106]. The spine of an animal functions similarly, with rigidly wound electrodes serving as the backbone to store energy, while flexible thin-film materials act as the marrow. Stacking these two components together achieves the overall deformability of the battery (Figure 9b) [107]. Bao et al. applied the bead-on-string structure design to flexible lithium batteries, quantitatively discussing the flexibility, energy density, and safety criteria of bead-on-string LIBs through analysis of each finite deformation model [10]. Due to the independent existence of the rigid part of the battery in the deformation process, the deformation of the prismatic structured flexible lithium battery mainly relies on the flexible connection, so the seamless bending cannot be achieved at this present, and there is still considerable research space.

### 4.2. Folded Structure Battery

Folding batteries take the form and structure of typical flexible lithium batteries and are then transformed and upgraded from traditional planar lithium batteries, meaning that their manufacturing technology is simple and mature [108]. The foldability of electrode materials and the design of battery structure play decisive roles in the preparation process of folded batteries.

At present, in the research of flexible foldable electrode materials, the research on flexible current collectors is relatively mature. In traditional LIBs, when using copper foil as the current collector, the active material is prone to detach from the current collector during bending and folding processes. Zhang et al. proposed a method of in situ growth of CuO nanorods on copper foil to enhance the adhesion of active materials as flexible electrodes (Figure 9c) [109]. The novel material CNT possesses excellent conductivity and flexibility, thus offering significant advantages in constructing flexible electrode current collectors [110]. The network structure of CNTs contains numerous pores and a large specific surface area, which not only improves the mechanical performance of the electrode but also allows better adhesion of the active materials and wetting by the electrolyte [111]. Compared to traditional lithium batteries, lithium batteries with multi-walled CNTs (MWNT) as current collectors (spinel-structured lithium titanate (Li_4_Ti_5_O_12_)//LiFePO_4_) exhibit a 14-fold reduction in voltage fluctuation under 4.2% bending strain; after 288 repeated folding cycles, the overall mechanical performance of the battery remains excellent [112]. Based on the method of enhancing structural resilience, insulation polymer substrates can be incorporated into the carbon nanomaterial-based current collector electrodes to reinforce the substrate and enhance the folding durability of flexible batteries [113].

At present, research on flexible batteries mostly focuses on the development of materials for individual cells. The design of flexible battery packs can significantly enhance battery energy density and durability. Using a multi-layer stacking approach, two positive electrodes are sandwiched on either side of the negative electrode within each individual battery cell, preventing short circuits by means of a separator (Figure 9d). During the bending and folding process, the textile-based electrodes on both sides can exhibit excellent flexibility by releasing counteracting stress [114]. Inspired by origami folding, Liao et al. proposed a flexible battery design achieved by connecting two adjacent energy storage segments through a serrated structure (Figure 9e). The battery can withstand extreme dynamic folding operations (130°, 45,000 times) while maintaining stable capacity at 1 C [115].

### 4.3. Helical Structured Flexible Battery

In addition to the above-mentioned typical types of flexible batteries, the design of helical structure flexible batteries has also attracted widespread attention. Meng et al. designed a flexible lithium battery inspired by the Deoxyribonucleic acid (DNA) double helix structure. This spiral-structured battery is mainly composed of multiple energy storage units and some stress-buffering dimples (Figure 9f) [116]. The battery of a single energy storage unit can use CNT fiber springs as electrodes, enabling the battery to possess self-supporting and stretchable properties [117]. After 9000 cycles, the spiral-structured lithium battery still maintains stable stretching durability and exhibits good voltage retention under 1300% stress deformation [11].

### 4.4. Cable-Type Batteries

In the flexible battery structure, flexible 1D batteries with unique advantages such as miniaturization, adaptability, and weaveability have prominent commercial application prospects. Among them, cable-based LIBs may provide a necessary breakthrough for wearable electronics due to their excellent mechanical flexibility [118]. Since the cable-based flexible battery has a coaxial structure, the battery components need to be completed under special processing and preparation techniques, such as 3DP and electrochemical deposition technology, so that the electrodes and electrolyte can be coaxial aligned. Zhao et al. used 3DP technology to prepare flexible Li_4_Ti_5_O_12_/rGO fiber electrodes by using reduced graphene-based printing inks. Due to the high specific surface area of the reduced rGO, a 3D conductive network is constructed inside the fiber electrode, which enhances the electronic conductivity and ion mobility. The prepared fiber electrode not only exhibits excellent mechanical properties (35% elongation at break) but also excellent electrochemical properties (83% capacity retention after 100 cycles) [77]. Similarly, Ling et al. used rapid ink extrusion to introduce pyrrole-modified reduced rGO, which led to the assembly of CNTs and poly(vinylidene fluoride-co-hexafluoropropylene) to form a robust, conductive, and agglomerate-free 3D network for linear loading of the active material. The assembled cable-type battery provides excellent cycling stability (93.9%/1 C/100 cycles) and highly stable power output (e.g., 94.8%/1 C/1000 bending cycles) in different deformation states [119].

In addition, cable-based LIBs usually use fibrous or two-dimensional carbon materials with good mechanical properties and continuous electronic conduction to prepare flexible electrodes so that the active materials can better adhere to the carbon materials to adapt to the deformation of cable-type batteries in practical applications [120]. Hoshide et al. prepared current collectors with excellent mechanical toughness by regular packing and in situ hybridization of two-dimensional titanium oxide nanosheets with reduced rGO. Compared to most fiber electrodes, this new electrode offers significant advantages: a high linear density of active material, maximum exposure of active sites, and a stacked configuration with nanochannels between sheet structures. These properties confer excellent mechanical flexibility and electrochemical properties on fiber batteries with new electrodes, including high linear capacity, superior rate capability, and vastly improved cycling stability [121].

Of course, the design of the flexible battery structure not only includes the flexible design of a single battery directly, but also the flexible interface mode for the unit battery, and the combination and integration of rigid and flexible batteries. The “cell-to-bod (CTB) structure battery” for electric vehicles is a new type of battery design and integration, in which the battery cells are directly integrated into the body or chassis structure [122,123]. In 2022, Build Your Dreams (BYD) released its CTB technology to address next-generation battery pack design and system-level integration needs. The battery pack is designed with a sandwich structure and consists of an upper cover, BYD’s signature blade battery cell, and an underbody protective disc, which significantly improves the integration level and increases the volume utilization rate to 66%. This integrated battery-body structure enhances overall structural strength and significantly reduces invasive damage under frontal, small overlap, and sidebar impacts [123].

**Table 4:** Study on flexible battery designed with different flexible structures.

Structure	Electrode Materials	Wear-and-Tear Life	Ultimate Deformation State	Reference
Joint-inspired structure	Graphite‖LiCoO_2_	Maintain a discharge capacity of 135.9 mAh g^−1^ without capacity fading with a torsional angle of 45° at 1 C	Torsional angle of 45°	[106]
Joint-inspired structure	Graphite‖LiCoO_2_	Discharge capacity remained over 94.3% after 100 cycles deformed from flat to flexed and twisted during cycling at 0.2 C	1000 torsional deformations of 90°	[107]
Folded structure	CuO‖Li_2_MnSiO_4_	A slight decrease in the discharge capacities after 100 cycles of bending	Bending radius of 20 mm	[109]
Folded structure	Graphite‖LiCoO_2_	Maintain 99.5% capacitance after 10 cycles after bent with a diameter of 2 cm for 1000 times	Bending diameter of 6 cm	[115]
Helical structure	Graphite‖LiFePO_4_	Capacity retention of 95.7% after 220 cycles under five times the spiral–conventional conversion	Spiral degree of 360°	[116]
Cable-type	LFP/rGO/CNT‖LTO/rGO/CNT	Maintain 96.1% capacity retention at 1 C after repetitive bending for 1000 cycles	Bending degree of 180°	[119]
Cable-type	Titanium oxide nanosheets/rGO	Voltage remained nearly unchanged both when straight and in various bending conditions	Bending degree over 90°	[121]

## 5. Summary and Prospect

This review provides a detailed overview of flexible batteries, covering aspects from the preparation and modification of battery materials to the fabrication processes of advanced flexible materials and to the structural design of flexible batteries. It discusses the key issues in realizing the preparation of flexible batteries. Although significant research progress has been made in flexible batteries, there remains considerable room for development in the commercialization of high-performance flexible lithium batteries. The future development of flexible batteries includes the following aspects:

(1) Integrated Design of Flexible Battery Materials. This can enhance the closeness of interlayer contact to reduce the negative issues arising from battery deformation. For example, the active material can be grown in situ on the surface of the current collector or be integrated with the current collector using a casting method. Therefore, in future, we expect to see more integrated and seamless design of electrodes, electrolytes, and package housings.
(2) Simplification and cost-effectiveness of battery processes. At present, the preparation process of flexible batteries includes the preparation of electrode and electrolyte materials and the packaging and assembly of batteries. These preparation processes will inevitably lead to the complexity of the preparation process and increase the operating cost of mechanical equipment. For example, many simple and easy-to-operate technologies such as coating and printing can be used to produce flexible battery materials on a large scale.
(3) Development of multifunctional flexible batteries. Flexible electronic products may be able to meet the demands of deformation in different directions, such as folding, bending, and stretching at the same time in the practical application process, but current flexible batteries, whether they are made using electrode material or the battery structure design, find it difficult to meet the needs of various deformations. Therefore, it is necessary to develop flexible batteries that can achieve multiple deformations.
(4) Establishing a comprehensive evaluation system for flexible batteries. Establish standards for flexible batteries from materials to performance and provide standards and comparison templates for researchers to develop flexible battery performance. It is mainly the mechanical propertiesand electrochemical performance during deformation of flexible electrode materials that require attention.

## Figures and Tables

**Table 1:** Comparison of the flexible LIBs with the traditional.

Component	Flexible LIBs	Traditional LIBs
Current collector	Flexible, lightweight and high adhesion current collectors, e.g., CNT coated cellulose paper	Metal foil current collector, e.g., Cu foil (for anode) or Al foil (for cathode) with a thickness of 10–30 μm.
Electrode active materials	Flexible, lightweight and composite electrode active materials, e.g., CNT-based anode and organic composite cathode	Anode: lithium metal, carbon-based materials, Si, metal oxide, etc. cathode: lithium metal oxide such as LiCoO_2_, LiFePO_4_, LiNi_0.8_Co_0.1_Mn_0.1_O_2_, etc. lithium-free oxide, sulfide
Binder	No binders	Polymer: PVDF, PTFE, PVA, etc.
Conductive additive	No conductive additives	Carbon black, CNTs, graphite, metal powder
Electrolyte	Inorganic ceramic electrolyte, polymer electrolyte	Liquid or solution (ionic liquid, carbonate-based lithium salt electrolyte and lithium salt gel electrolyte)
Separator	Commonly no separator	Polymer separator: PP, PE, PVDF, PVC etc.
Package and Shape	Polymer package, pouch type	Stainless steel package, aluminum or aluminum plastic seal, cylindrical, square, pouch, plate, coin type

PVDF: polyvinylidene fluoride; PTFE: polytetrafluoroethylene: PVA: polyvinyl alcohol; PP: polypropylene; PE: polyethylene; PVC: polyvinyl chloride.

**Table 2:** Comparison of different kinds of flexible carbon-based materials.

Carbon-Based Materials	Synthesis Methods	Flexibility	Electrical Conductivity	Battery Performance	Ref.
MnO_x_/MWCNTs nanocomposites	Hydrothermal method combined with thermal treatment	Moderate	Moderate	1353.2 mAh g^−1^ at 0.1 A g^−1^ and 664.8 mAh g^−1^ after 1000 cycles at 2 A g^−1^	[24]
C@Fe_2_O_3_/SWCNT membrane	FCPEF method followed by annealing treatment	Moderate	High	1294.7 mAh g^−1^ at 50 mA g^−1^; 563.7 mAh g^−1^ followed at 2 A g^−1^ after 200 cycles	[29]
Ni_x_Co_y_-silicate@CNTs film	Floating catalyst chemical vapor deposition (FCCVD), hydrothermally treatment	Good	High	1047 mAh g^−1^ at 100 mA g^−1^	[30]
LFP/CNT/CNF and LTO/CNT/CNF	Directional freezing assisted 3DP technology	Excellent	High	8.4 mAh cm^−2^ at 0.2 C and h capacity retention rate of 59.1% from 0.2 to 5 C of the 8-layer full cell	[4]
CNTs coated cellulose-based paper	Additive-free roll-to-roll spray coating technique	Moderate	Moderate	A high energy density of 460 W h kg^−1^ at a power density of 250 W kg^−1^	[34]
TiO_2_ nanorods/rGO composites	Sol-static self-assembled method followed by alkali-heat treatment	Moderate	High	250.1 mA h g^−1^ at 2 A g^−1^; 353.6 mA h g^−1^ after 100 cycles at 0.1 A g^−1^	[39]
Fe_2_VO_4_/rGO composite	Solvothermal treatment and subsequent calcination	Moderate	High	223.6 mAh g^−1^ at 10 A g^−1^ after 2500 cycles	[40]
NMC811-Graphene	Solid-state method of simple direct mixing	Moderate	Moderate	Capacity retention rate of 81.32% at 1 C after 1000 cycles	[43]
BDT/3DGraphene composite	Impregnation method	Moderate	Moderate	100 mAh g^−1^ at 4 C	[44]
Graphene foil	Blade-coating method followed by high temperature annealing	Moderate	High	98.0, 59.3 and 42.0 mAh g^−1^ at 0.5 C, 2.0 C and 5.0 C after 200 cycles	[45]
Multishell CF/ECF/NiO/CD	Hydrothermal method; thermal treatment	Moderate	High	40.89 mA h cm^−2^ (retaining 98%) after 300 cycles	[47]
LiFePO_4_/EDOT/CFs	Low temperature pyrolysis treatment, one-pot process	Moderate	High	129 mAh g^−1^ at 0.2 C	[48]
Cu/Cu_3_P-N-CNFs	Electrospinning	Moderate	Moderate	568 mAh g^−1^ at 2 C of Li@Cu/Cu_3_P-N-CNFs||S@CNTs full cell	[49]
C@IENiOCC	Ion exchange	Excellent	High	3.08 mAh cm^−2^ at 0.25 mA cm^−2^ and 1.78 mAh cm^−2^ at 8 mA cm^−2^	[14]
NiP_2_@NC	Hydrothermal reaction, calcination, carbothermal reduction	Moderate	High	150.1 mAh g^−1^ and capacity retention of 87.4% after 100 cycles at 0.1 A g^−1^	[51]

**Table 3:** Advantages and disadvantages of different preparation methods.

Preparation Process	Advantages	Disadvantages
3D printing	Capacity to fabricate highly accurate 3D structures with micrometer resolution, excellent conformal deposition and good mechanical flexibility	Limited ink materials available for 3DP
Electrospinning technology	Enables the preparation of materials with high porosity, simple and flexible operation, wide range of applications	Low production efficiency, high cost, the use of organic solvents easy to cause environmental pollution
Coating	Low cost, easy scalability and good substrate selectivity	Thickness limitation, insufficient adhesion, solvent residue
Electrophoretic deposition	Good retention of physical and chemical properties, controlled thickness, uniform coating	High equipment cost, insufficient adhesion, precise control of voltage and time required
Electrodeposition	Fast deposition rate, controlled deposition thickness	High cost, difficulty of preparing thin-film materials with complex compositions
Physical vapor deposition	Relatively low deposition temperature, consistent compositional transfer, no limitation on target material.	Poor uniformity, low adaptability to complex shapes
Chemical vapor deposition	Ability to deposit on irregular surfaces with good conformality, fast deposition rate	Toxicity and limitedavailability of precursors
